# Transcriptomic differentiation underlying marine‐to‐freshwater transitions in the South American silversides *Odontesthes argentinensis* and *O. bonariensis* (Atheriniformes)

**DOI:** 10.1002/ece3.3133

**Published:** 2017-06-07

**Authors:** Lily C. Hughes, Gustavo M. Somoza, Bryan N. Nguyen, James P. Bernot, Mariano González‐Castro, Juan Martín Díaz de Astarloa, Guillermo Ortí

**Affiliations:** ^1^ Department of Biological Sciences The George Washington University Washington DC USA; ^2^ Instituto de Investigaciones Biotecnológicas‐Instituto Tecnológico de Chascomús (CONICET‐UNSAM) Chascomús Argentina; ^3^ Consejo Nacional de Investigaciones Científicas y Técnicas (CONICET) Buenos Aires Argentina; ^4^ Computational Biology Institute The George Washington University Washington DC USA; ^5^ Institute for Biomedical Sciences The George Washington University Washington DC USA; ^6^ Grupo de Biotaxonomía Morfológica y molecular de peces IIMyC‐CONICET Universidad Nacional de Mar del Plata Mar del Plata Argentina

**Keywords:** ecological genetics, fish, speciation, transcriptomics

## Abstract

Salinity gradients are critical habitat determinants for freshwater organisms. Silverside fishes in the genus *Odontesthes* have recently and repeatedly transitioned from marine to freshwater habitats, overcoming a strong ecological barrier. Genomic and transcriptomic changes involved in this kind of transition are only known for a few model species. We present new data and analyses of gene expression and microbiome composition in the gills of two closely related silverside species, marine *O. argentinensis* and freshwater *O. bonariensis* and find more than three thousand transcripts differentially expressed, with osmoregulatory/ion transport genes and immune genes showing very different expression patterns across species. Interspecific differences also involve more than one thousand transcripts with nonsynonymous SNPs in the coding sequences, most of which were not differentially expressed. In addition to characterizing gill transcriptomes from wild‐caught marine and freshwater fishes, we test experimentally the response to salinity increases by *O. bonariensis* collected from freshwater habitats. Patterns of expression in gill transcriptomes of *O. bonariensis* exposed to high salinity do not resemble *O. argentinensis* mRNA expression, suggesting lack of plasticity for adaptation to marine conditions in this species. The diversity of functions associated with both the differentially expressed set of transcripts and those with sequence divergence plus marked microbiome differences suggest that multiple abiotic and biotic factors in marine and freshwater habitats are driving transcriptomic differences between these species.

## 1 INTRODUCTION

The marine–freshwater boundary is a strong ecological gradient for aquatic organisms across the tree of life. Ray‐finned fish lineages have transitioned from marine to freshwater multiple times in their evolutionary history, more frequently than in the opposite direction (Betancur‐R, Ortí, & Pyron, 2015), repeatedly adapting to meet this ecological challenge. There are many obstacles for the successful colonization of freshwater habitats by marine fishes, and the most physiologically demanding is the salt concentration in the water. Most fishes are stenohaline (have narrow salinity tolerance), and just 2% of all fishes are euryhaline, occupying both marine and freshwater environments (Betancur‐R et al., 2015).

Maintaining osmotic homeostasis is a major challenge for organisms that move between marine and freshwater habitats. Osmoregulation in fresh water is energetically expensive for marine fish, whereas sea water is closer to the internal solute concentration of fishes, requiring less energy to maintain homeostasis (Lee & Bell, 1999). Coping with the salinity concentration gradient, however, may not be the main or only challenge. Other abiotic impacts include the relative lack of calcium available in fresh water, critical because teleosts absorb calcium for bone formation directly from the surrounding waters (Bell, Orti, Walker, & Koenings, 1993; Simmons, 1971). Temperature fluctuations are greater in freshwater habitats, presenting less opportunity to escape inhospitable temperatures (Lee & Bell, 1999). Biotic factors also are likely to be important, given that microbial communities are strongly structured by salinity (Logares et al., 2009; Lozupone & Knight, 2007), and fish must tolerate drastic challenges to their immune system as they experience complete turnover of their microbiomes (Lokesh & Kiron, 2016; Schmidt, Smith, Melvin, & Amaral‐Zettler, 2015) or interactions with novel parasites. These ecological differences often drive genetic divergence between closely related marine and freshwater organisms.

Genomes are shaped by an organism's ecology (Ungerer, Johnson, & Herman, 2008), and fish species and populations that transition from marine to fresh water offer important insight into how this occurs. For example, significant divergence of SNPs associated with freshwater adaptation has been detected in or near genes related to osmoregulation, stress, and immune function by comparing marine and freshwater populations of sticklebacks and killifishes (Hohenlohe et al., 2010; Jones et al., 2012; Kozak, Brennan, Berdan, Fuller, & Whitehead, 2013). As many of these habitat transitions may lead to speciation, detecting and characterizing transcriptomic divergences may be of importance to better understand this process, as patterns of gene expression can evolve rapidly (Wolf et al., 2010). Osmoregulatory genes have population and species‐specific expression patters in fundulid killifishes inhabiting different salinities (Kozak et al., 2013; Whitehead, Galvez, Zhang, Williams, & Oleksiak, 2011; Whitehead, Roach, Zhang, & Galvez, 2012), and transcriptional responses to temperature are more variable in freshwater than marine stickleback (Morris et al., 2014). Furthermore, marked and predictable differences in microbiome composition have been reported to follow acclimation of the black molly (*Poecilia sphenops*) to different water salinity, suggesting a key role of the immune system in habitat transitions (Schmidt et al., 2015).

Here, we investigate adaptation in gill function following marine‐to‐freshwater invasions by presenting new data and analyses of transcriptomes and microbiomes obtained from gills of silverside fishes of the genus *Odontesthes*. If ecology is driving speciation between marine and freshwater silversides, we would expect fixed genetic differences and diverging patterns of gene expression between fishes occupying these two habitats, notably for genes related to salinity tolerance, calcium intake, temperature fluctuation, and the immune system. The combined challenges presented by abiotic (salinity, temperature) and biotic (microbiome composition) factors may lead to complex interactions driving genomic and transcriptomic differentiation between habitats. Alternatively, if expression differences are plastic responses to habitat transitions, common garden experiments would reveal similar expression patterns for marine and freshwater species under the same conditions.

We study *Odontesthes* silversides that comprise 19 marine and freshwater species, commonly known as pejerreyes (Dyer, 2006), which have recently and frequently transitioned from marine to freshwater in southern South America (Bloom, Weir, Piller, & Lovejoy, 2013; Campanella et al., 2015). They are members of the order Atheriniformes, whose members inhabit coastal marine and freshwater environments worldwide, having successfully and independently colonized freshwater habitats on multiple continents (Campanella et al., 2015). Plastic responses to the environment observed in some estuarine atherinid species are hypothesized to be a pre‐adaptation for successfully undertaking transitions to fresh water (Bamber & Henderson, 1988), and species or species complexes that inhabit different salinity gradients often show genetic structuring and evidence for incipient speciation (Beheregaray & Sunnucks, 2001; Fluker, Pezold, & Minton, 2011; Kraitsek, Klossa‐Kilia, Papasotiropoulos, Alahiotis, & Kilias, 2008). We focus on a pair of species, one marine and one freshwater: *O. bonariensis*, native to lakes and rivers of the Pampas region of Argentina, the Rio de la Plata and Lower Paraná and Uruguay basins, up to the Tramandaí coastal lagoon system in Brazil (Dyer, 2006; Liotta, 2005; Somoza et al., 2008); and *O. argentinensis,* is its marine (putative) sister species that ranges along the South Atlantic coast of Buenos Aires Province (Argentina) to Southern Brazil (Campanella et al., 2015; Dyer, 2006). Up to eight, sometimes sympatric, freshwater *Odontesthes* species have been described in this region, associated with rapid divergence in freshwater habitats as a result of recent changes in sea level (Beheregaray, Sunnucks, & Briscoe, 2002). All these freshwater species are closely related to *O. argentinensis* and *O. bonariensis*.

Whether due to rapid speciation in freshwater habitats or ongoing gene flow among incipient freshwater species and marine fishes, there is little genetic differentiation among species in the *argentinensis‐bonariensis* species complex (Campanella et al., 2015; Díaz et al., 2016; García et al., 2014; González‐Castro, Rosso, Mabragaña, & Díaz de Astarloa, 2016). *Odontesthes argentinensis* and *O. bonariensis* show patterns of genetic divergence that fall well within the range of intraspecific variation (Heras & Roldán, 2011), but they differ in some meristic counts of morphological characters and their body shape (Dyer, 2006; González‐Castro et al., 2016), and their mechanism of sex‐determination appears to be different (Fernandino, Hattori, Strussmann, & Somoza, 2013; Strussmann, Saito, Usui, Yamada, & Takashima, 1997; Yamamoto, Zhang, Sarida, Hattori, & Strüssmann, 2014). One of the most conspicuous differences between the two species is the different habitats they occupy.

Considering the substantial ecological boundary that the marine‐to‐freshwater transition poses for fishes but lack of known genetic divergence between these two species, we investigate underlying changes at the genomic level associated with freshwater adaptation in these species by comparing gill transcriptomes from wild‐caught *O. argentinensis* (marine) and *O. bonariensis* (freshwater). We also test for phenotypic plasticity in gene expression by comparing gill transcriptomes from laboratory‐housed *O. bonariensis*, which is still relatively euryhaline (Tsuzuki, Strüssmann, & Takashima, 2008; Vigliano, Aleman, Quiroga, & Nieto, 2006), in freshwater and brackish salinities. Adaptive genetic divergence can take place by mutations in the coding sequences or by changes to *cis*‐regulatory elements that affect expression, though often changes are the result of both (Hoekstra & Coyne, 2007). The teleost gill is of critical physiological importance through its direct interaction with the environment, fulfilling respiratory, osmoregulatory, and immune functions (Díaz, Castro, García, Díaz de Astarloa, & Figueroa, 2009; Evans, 2005), and genes of adaptive significance in marine or fresh water are likely to be expressed in this tissue, which could be implicated in speciation between *O. argentinensis* and *O. bonariensis*. We provide new data to characterize diverging patterns of gene expression and genetic differences associated with functions related to freshwater adaptation, including ion transport, calcium uptake, temperature acclimation, and the immune system. We also experimentally test whether the pattern of gene expression is a plastic response to the environment. Finally, we characterize the gill microbiomes of *O. bonariensis* and *O. argentinensis*, which are predicted to diverge along the water salinity gradient, hence providing a baseline for understanding putative changes in immune‐response transcripts in the gills.

## 2 METHODS

### 2.1 Sampling and experimental design

**Figure 1:**
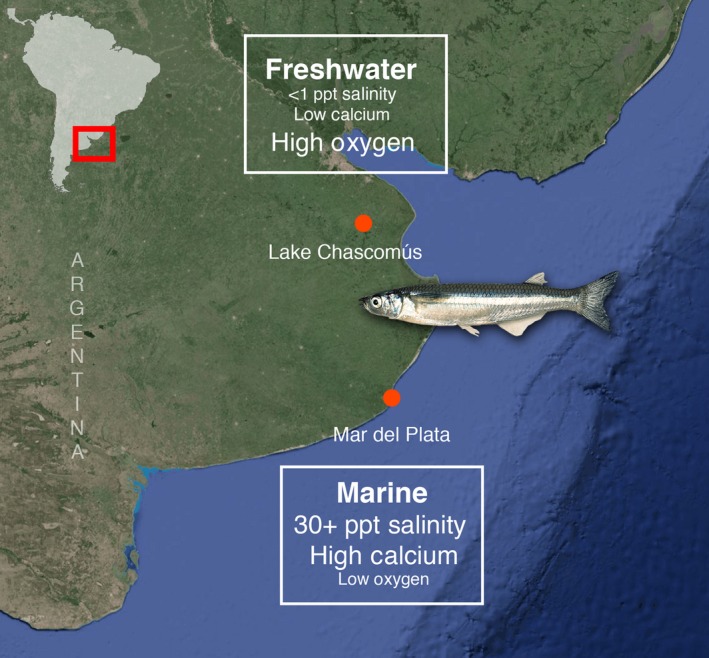
Sampling localities for *O. argentinensis* at Mar del Plata, and *O. bonariensis* at Lake Chascomús, Argentina, with abiotic factors that are typically different between marine and freshwater environments

We obtained gill tissue from three individuals of *O. argentinensis* collected by beach seining along the Atlantic coast near Mar del Plata (38°02′S, 57°31′W) on March 3, 2014, and three *O. bonariensis* fished by trawl from Lake Chascomús (35°34′S, 58°01′W) on March 10, 2014, in Buenos Aires Province, Argentina (Figure 1). Natural salinity in this lake is about 2 ppt. Gills were immediately preserved in RNA*later* ^®^ in the field, kept at ambient temperature, and promptly transferred to −80°C in the lab until RNA extraction. An experiment to test the effect of increasing salinity was set up with an additional six adult *O. bonariensis* collected from Lake Chascomús. These fish were transferred to nearby freshwater tanks at IIB‐INTECH (Chascomús) with recirculating water at 18°C and kept under natural photoperiod. There, three silversides were placed in a second tank where salinity was increased over two days to 15 ppt salinity. This is not beyond the salinity tolerance for this relatively euryhaline species, which is in fact known to have reduced cortisol levels at 20 ppt salinity compared to 0 ppt waters (Tsuzuki, Ogawa, Strüssmann, Maita, & Takashima, 2001). Fish were allowed to acclimate for five days, before they were sacrificed with an MS‐222 overdose, and gill tissue was collected. All procedures followed the UFAW Handbook on the care and management of laboratory animals (http://www.ufaw.org.uk) and internal IIB‐INTECH institutional regulations. Tissues were stored in RNA*later* ^®^ at −80°C until RNA was extracted using a Qiagen RNeasy kit, following manufacturer's instructions.

### 2.2 RNA sequencing

Messenger RNA (mRNA) was isolated from total RNA using a Dynabeads^®^ mRNA purification kit (Ambion). We prepared paired‐end Illumina sequencing libraries for each gill sample with a SMARTer Stranded RNA‐seq kit (Clontech). Resulting cDNA libraries were quantified via qPCR with the KAPA Library Quantification kit (KapaBiosystems). We sequenced 92‐bp paired‐end reads on four lanes of an Illumina HiScan, with two separate runs at Children's National Medical Center in Washington, DC. Samples were multiplexed and sequenced together across all lanes. Raw sequences for all samples in this project are accessible at NCBI (BioProject PRJNA311227).

### 2.3 Transcriptome assembly

Bioinformatics analyses were performed on the Colonial One high performance computing system at George Washington University. Raw Illumina base calls were converted to FASTQ‐formatted files and demultiplexed with CASAVA v1.8.2 (Illumina). These reads were deposited in the SRA database with accession numbers noted in Table 1. We used Trimmomatic v0.33 (Bolger, Lohse, & Usadel, 2014) to quality control our sequences and remove adapter contamination using ILLUMINACLIP:2:30:7 HEADCROP:13 SLIDINGWINDOW:7:15 MINLEN:20 as trimming parameters. Contaminating ribosomal RNA (rRNA) sequences were removed with SortMeRNA v2.0 (Kopylova, Noe, & Touzet, 2012). Paired‐end reads from all samples were mapped against the *O. bonariensis* genome (Campanella, 2014) using STAR v2.5 (Dobin et al., 2013), and assembled using Trinity v2.1.1 in genome‐guided mode. Open reading frames (ORFs) were predicted using TransDecoder v2.0.1 (Grabherr et al., 2011; Haas et al., 2013). We collapsed identical transcripts with ORFs using the program CD‐HIT v4.6, retaining the longest transcripts (Fu, Niu, Zhu, Wu, & Li, 2012; Li & Godzik, 2006). We functionally annotated transcripts by blasting protein sequences against NCBI's Non‐Redundant (nr) protein database, accessed June 1st, 2016, and mapping gene ontology terms using Blast2GO Basic v3.2 software.

**Table 1:** Gill Transcriptomes of *Odontesthes argentinensis* and *O. bonariensis* and their associated NCBI database accession numbers

Species	Treatment	Filtered Reads (PE)	SRA Accessions
*O. argentinensis*	Wild, Mar del Plata	4,457,408	SRX1671790
*O. argentinensis*	Wild, Mar del Plata	5,198,031	SRX1681012
*O. argentinensis*	Wild, Mar del Plata	3,364,197	SRX1681017
*O. bonariensis*	Wild, Lake Chascomús	2,371,170	SRX1681471
*O. bonariensis*	Wild, Lake Chascomús	7,908,074	SRX1681473
*O. bonariensis*	Wild, Lake Chascomús	1,930,665	SRX1681474
*O. bonariensis*	Laboratory, 0 ppt salinity	2,028,758	SRX1681475
*O. bonariensis*	Laboratory, 0 ppt salinity	6,504,388	SRX1681516
*O. bonariensis*	Laboratory, 0 ppt salinity	2,093,475	SRX1681556
*O. bonariensis*	Laboratory, 15 ppt salinity	118,799	SRX1681557
*O. bonariensis*	Laboratory, 15 ppt salinity	6,099,175	SRX1681558
*O. bonariensis*	Laboratory, 15 ppt salinity	4,324,427	SRX1681559

### 2.4 Differential expression and gene set enrichment analyses

**Figure 2:**
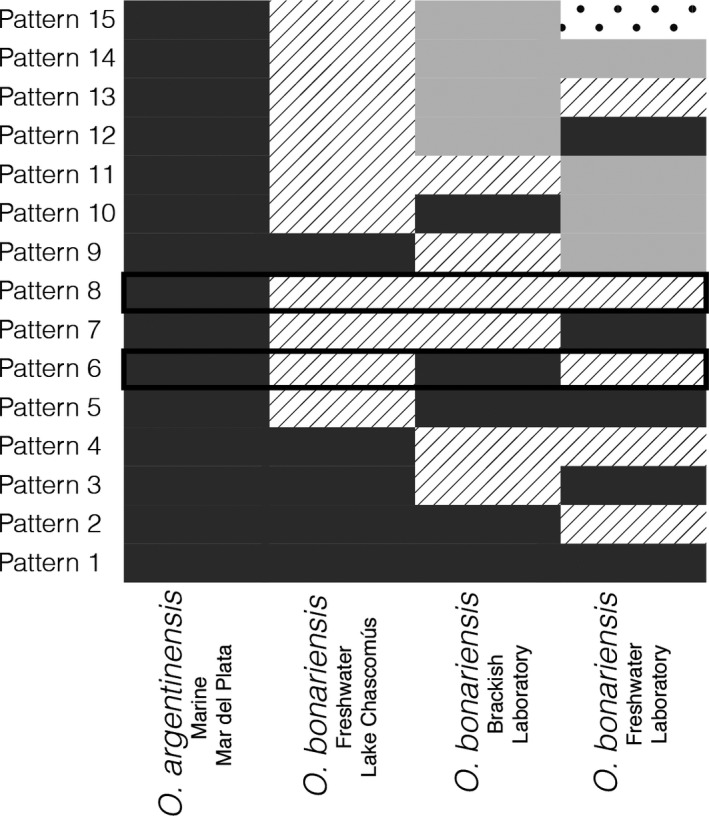
Expression condition comparisons for four treatments in Ebseq. Patterns discussed in the text are highlighted with black boxes, including the pattern that differentiates the different species (pattern 8), and the pattern that differentiates higher from lower salinity treatments (pattern 6). Pattern 8, which differentiates the marine *O. argentinensis* treatment, was detected for 3,271 transcripts, while only 96 were detected for pattern 6. For 230 transcripts, pattern 4 was the best fit, suggesting some stress in laboratory conditions

We used assembled transcripts with ORFs to test for differential expression among gill transcriptomes of wild specimens obtained from marine and freshwater habitats, as well as the two experimental lots (freshwater and brackish water tanks). Sequence reads were mapped for each individual sample back to the reference sequences with Bowtie2 (Langmead & Salzberg, 2012). Transcript abundance was estimated using the Trinity wrapper for RSEM (Li & Dewey, 2011). We quantified expression patterns using the R package EBSeq, which allows for testing different expression patterns when the experimental design has more than two treatments (Leng et al., 2013). We tested all 15 possible expression patterns for four treatments (wild‐marine, wild‐lake, freshwater‐laboratory, brackish‐laboratory; Figure 2), and ran EBSeq with 50 iterations. We considered transcripts with a posterior probability >.95 for a particular expression to be differentially expressed, which corresponds to an FDR of 0.05.

Gene ontology (GO) terms were mapped using Blast2GO Basic software. We used Fisher's exact test to determine which biological processes (GO terms) were enriched among differentially expressed genes from *O. argentinensis* from any *O. bonariensis* treatment, as well as those transcripts that were similarly differentially expressed in *O. argentinensis* and *O. bonariensis* at brackish salinity.

### 2.5 SNP calling

We followed the protocol for SNP detection on transcriptome assemblies described by De Wit et al. (2012). We used Picard‐tools v1.129 to coordinate sort, add read group names, and mark suspected duplicate reads in our SAM files obtained from Bowtie2 for each of our separate individuals, and to index and merge these into a single BAM file for SNP discovery. We used the recommendations of De Wit et al. (2012) to run the Genome Analysis Toolkit v3.4‐46 for SNP calling (McKenna et al., 2010). We used only those SNPs that were completely differentiated between marine and freshwater samples and fell in ORFs for further analysis, and had been genotyped for all individuals. We then examined whether these SNPs resulted in synonymous or nonsynonymous changes in the coding sequence of these transcripts, and pulled annotation information from our blastp output.

### 2.6 Microbiome characterization

For each individual gill sequenced we characterized the associated microbiome using the following steps. Filtered reads greater than 36 bp were error‐corrected with BLESS v0.22 using default settings (Heo, Wu, Chen, Ma, & Hwu, 2014). Reads matching conserved, informative loci were taxonomically assigned with PhyloSift (Darling et al., 2014) using the default settings and core marker set (version 1413946442), but excluding the 18S_rep marker. The matching reads were phylogenetically placed using Pplacer (Matsen, Kodner, & Armbrust, 2010). The 16S marker analysis was further used to quantify phylogenetic differences between samples with the Kantorovich–Rubinstein distance function in Pplacer, which is functionally similar to the weighted UniFrac metric (Evans & Matsen, 2012), and further explored using a phylogenetic edge PCA (Matsen & Evans, 2013) generated with the guppy package in Pplacer. Taxonomic diversity plots based on 16S identifications were generated in R using the phyloseq package (McMurdie & Holmes, 2013; Team, 2014).

## 3 RESULTS

### 3.1 Sequencing and transcriptome assembly

Sequences generated for each of our biological replicates are reported in Table 1, along with SRA accession numbers. Trinity, using the genome‐guided mode, assembled 100,845,415 bases into 168,570 transcripts, with an N50 of 838. Only 40,384 nonredundant transcripts contained open reading frames detected using Transdecoder, which was the set of transcripts we used to map reads from our replicates to assess differential expression. One replicate from our laboratory‐brackish water treatment had significantly fewer reads, and this replicate was not included in differential expression analysis, although these reads were included in the assembly. There is no difference in mapping *O. argentinensis* RNA‐seq reads back to the *O. bonariensis* reference genome, thus any transcript expressed by *O. argentinensis* and not *O. bonariensis* still exists in the *O. bonariensis* genome.

### 3.2 Differential expression, SNP detection, and gene ontology enrichment

A total of 3,271 transcripts were differentially expressed between *O. argentinensis* and any *O. bonariensis* treatment (Pattern 8, Figure 2). These transcripts were enriched for Gene Ontology biological process categories that included several functions related to ion transport including ion transmembrane transport, chloride transmembrane transport, and hydrogen transmembrane transport (Table 2). The GO term “immune system process” was also enriched for this differentially expressed set. Far fewer transcripts (96) in *O. bonariensis* show a plastic response to salinity, that is similar to wild‐marine *O. argentinensis* (Pattern 6, Figure 2). This set of transcripts was not enriched for any biological process, although it did contain transcripts that have osmosensing functions, including NEDD4 ubiquitin ligase (Fiol & Kültz, 2007).

**Table 2:** Over‐represented biological process Gene Ontology terms for differentially expressed transcripts between *Odontesthes argentinensis* and all *O. bonariensis* treatments

GO term	Description	FDR	*p*‐Value
GO:0015031	Protein transport	1.65E−05	4.37E−09
GO:0045184	Establishment of protein localization	1.65E−05	4.96E−09
GO:0071702	Organic substance transport	2.03E−05	9.15E−09
GO:0033036	Macromolecule localization	9.93E−05	5.98E−08
GO:0008104	Protein localization	1.34E−04	1.01E−07
GO:0006886	Intracellular protein transport	2.53E−04	2.29E−07
GO:0098660	Inorganic ion transmembrane transport	7.12E−04	7.50E−07
GO:0044283	Small molecule biosynthetic process	2.21E−03	2.66E−06
GO:0034613	Cellular protein localization	2.24E−03	3.37E−06
GO:0070727	Cellular macromolecule localization	2.24E−03	3.37E−06
GO:1901657	Glycosyl compound metabolic process	2.45E−03	4.05E−06
GO:0046034	ATP metabolic process	3.41E−03	6.16E−06
GO:0006810	Transport	4.22E−03	9.33E−06
GO:0046907	Intracellular transport	4.22E−03	9.34E−06
GO:0009199	Ribonucleoside triphosphate metabolic process	4.22E−03	9.52E−06
GO:0009119	Ribonucleoside metabolic process	4.48E−03	1.08E−05
GO:0051649	Establishment of localization in cell	5.28E−03	1.35E−05
GO:0009116	Nucleoside metabolic process	5.42E−03	1.59E−05
GO:0015672	Monovalent inorganic cation transport	5.42E−03	1.64E−05
GO:1901659	Glycosyl compound biosynthetic process	5.42E−03	1.66E−05
GO:0009141	Nucleoside triphosphate metabolic process	5.42E−03	1.71E−05
GO:0009161	Ribonucleoside monophosphate metabolic process	6.15E−03	2.04E−05
GO:0051234	Establishment of localization	6.29E−03	2.18E−05
GO:0006364	rRNA processing	6.33E−03	2.29E−05
GO:0009123	Nucleoside monophosphate metabolic process	6.70E−03	2.52E−05
GO:0009205	Purine ribonucleoside triphosphate metabolic process	6.85E−03	2.69E−05
GO:0006818	Hydrogen transport	6.85E−03	2.89E−05
GO:0015992	Proton transport	6.85E−03	2.89E−05
GO:0009144	Purine nucleoside triphosphate metabolic process	6.92E−03	3.02E−05
GO:0016072	rRNA metabolic process	7.62E−03	3.44E−05
GO:0044711	Single−organism biosynthetic process	8.15E−03	3.93E−05
GO:0009126	Purine nucleoside monophosphate metabolic process	8.15E−03	4.05E−05
GO:0009167	Purine ribonucleoside monophosphate metabolic process	8.15E−03	4.05E−05
GO:0042278	Purine nucleoside metabolic process	1.07E−02	5.69E−05
GO:0046128	Purine ribonucleoside metabolic process	1.07E−02	5.69E−05
GO:0002376	Immune system process	1.07E−02	5.91E−05
GO:0050686	Negative regulation of mRNA processing	1.07E−02	6.27E−05
GO:0033119	Negative regulation of RNA splicing	1.07E−02	6.27E−05
GO:0048025	Negative regulation of mRNA splicing, via spliceosome	1.07E−02	6.27E−05
GO:0051641	Cellular localization	1.20E−02	7.20E−05
GO:0034220	Ion transmembrane transport	1.53E−02	9.44E−05
GO:1902476	Chloride transmembrane transport	1.62E−02	1.03E−04
GO:0098662	Inorganic cation transmembrane transport	1.96E−02	1.27E−04
GO:0051179	Localization	1.98E−02	1.33E−04
GO:0098655	Cation transmembrane transport	1.98E−02	1.34E−04
GO:0009117	Nucleotide metabolic process	2.17E−02	1.50E−04
GO:1902600	Hydrogen ion transmembrane transport	2.34E−02	1.66E−04
GO:0006753	Nucleoside phosphate metabolic process	2.86E−02	2.07E−04
GO:0042147	Retrograde transport, endosome to Golgi	3.51E−02	2.66E−04
GO:0015991	ATP hydrolysis coupled proton transport	3.51E−02	2.70E−04
GO:0015988	Energy coupled proton transmembrane transport, against electrochemical gradient	3.51E−02	2.70E−04
GO:1905097	Regulation of guanyl−nucleotide exchange factor activity	3.63E−02	2.91E−04
GO:2001106	Regulation of Rho guanyl−nucleotide exchange factor activity	3.63E−02	2.91E−04
GO:1901135	Carbohydrate derivative metabolic process	3.63E−02	2.95E−04
GO:0042254	Ribosome biogenesis	3.73E−02	3.09E−04
GO:0006091	Generation of precursor metabolites and energy	4.64E−02	3.92E−04
GO:0090662	ATP hydrolysis coupled transmembrane transport	4.99E−02	4.28E−04

A total of 1,417 transcripts have nonsynonymous SNPs putatively fixed between the marine and freshwater samples. A minority (201) of these transcripts are also differentially expressed between *O. argentinensis* and all *O. bonaeriensis* treatments. The set of transcripts with nonsynonymous SNPs were not enriched for any biological process GO terms.

### 3.3 Microbiome characterization

**Figure 3:**
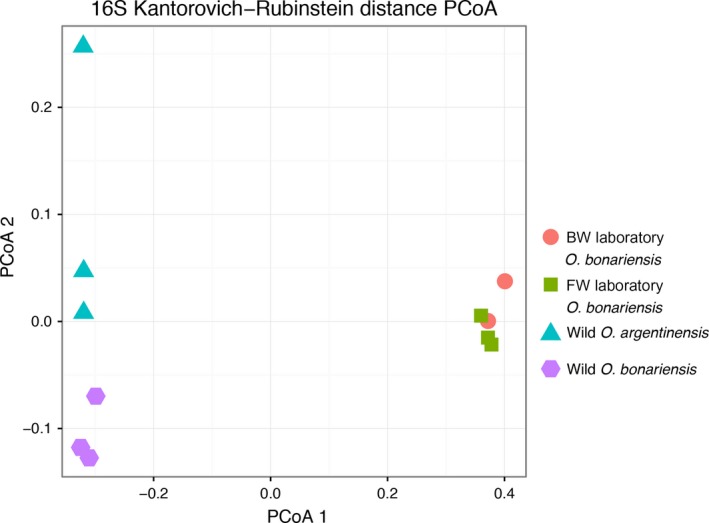
Principal coordinate analysis using Kantorovich‐Rubenstein distance comparing bacterial gill communities. Identification of microbial taxa was based on the 16S marker, using the PhyloSift pipeline

**Figure 4:**
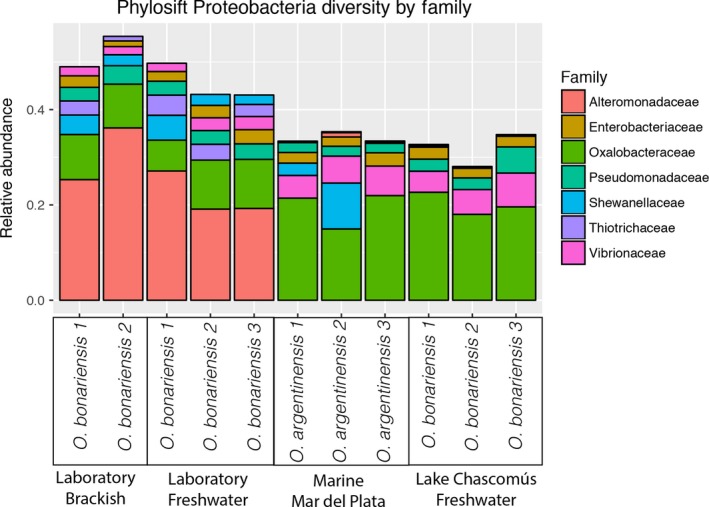
Relative abundance of Proteobacteria families detected from gill samples, identified via PhyloSift. Because samples are RNA, relative abundance represents the confounded variables of microbe presence and microbial gene expression

There was substantial variation in the composition of bacterial gill microbiota among wild‐caught individuals but far less among laboratory treatments (Figure 3). Although there is some differentiation between wild‐caught freshwater and marine gill microbiomes, laboratory‐housed fish displayed the most distinct microbiome. This characteristic laboratory microbiome was not substantially changed by increasing water salinity to 15 ppt. Laboratory individuals were caught in the wild along with those individuals from Lake Chascomús, and so changes in their microbiome likely occurred during the week‐long period, while they were housed at IIB‐INTECH. Phylogenetic edge PCA results suggest that a substantial amount of variation between samples is explained by comparing wild specimens with fish being housed in the laboratory (see supplemental files on Figshare). While all bacterial communities were dominated by Proteobacteria, gills collected from laboratory individuals had a large proportion of bacterial reads assigned to taxa from the family Alteromonadaceae, which was uncommon in wild individuals of either *O. argentinensis* or *O. bonariensis* (Figure 4).

## 4 DISCUSSION

### 4.1 Divergence between marine and freshwater fishes

The genetic data (mitochondrial DNA and few nuclear gene sequences) available to compare *O. argentinensis* and *O. bonariensis* prior to our transcriptome analysis suggested very little genetic divergence, within the typical boundaries of intraspecific variation (Díaz et al., 2016; García et al., 2014; González‐Castro et al., 2016; Heras & Roldán, 2011). The transcriptomic differences reported here likely reflect rapid speciation and adaptation to different environmental conditions, while still failing to accumulate the level of genetic differentiation in mitochondrial markers that are sometimes used to distinguish species.

The most significant pattern of differentially expressed genes separates *O. argentinensis* from all *O. bonariensis* samples. This suggests that habitat differences are potentially driving divergent patterns of expression between these two species. Our study detected differential expression of many candidate genes known to be involved in osmoregulation and ion permeability in fish gills, putatively ecologically important for the transition from marine to freshwater. As predicted, many transcripts that were differentially expressed between the two species were enriched for gene ontology terms related to ion transport (Table 2), as major physiological changes need to occur in order to maintain internal solute concentrations in these different environments. Although our sample sizes are relatively small to consider these differences at face value to represent fixed species‐level differences (Conesa et al., 2016), our results reveal consistent changes predicted on the basis of gill function adapting to different habitats.

Also based on a limited number of individuals (three marine, eight freshwater), we discovered 1,417 transcripts carrying nonsynonymous differences in coding sequences that are putatively fixed between *O. argentinensis* and *O. bonariensis*. With deeper sampling of individuals, however, many of these differences may turn out to be shared between these species. Furthermore, many of these differences are likely neutral or may have no significant adaptive advantages for these environments. In any case, we consider this list of candidate genes worth investigating, as it contains some well‐known osmoregulatory genes in fishes that likely are under selection to adapt to different habitats, including Na/K transporting ATPase subunit alpha 1. This ion transporter—one of the most well‐known osmoregulatory genes in fish—is both differentially expressed between *O. argentinensis* and all *O. bonariensis* treatments, and contains a nonsynonymous SNP. Variants of this gene have been linked to freshwater transitions in the stickleback system (Jones et al., 2012). Aquaporin 3 also contained nonsynonymous SNPs, a gene thought to be adaptive for osmoregulation in sticklebacks, tilapia, and killifishes (Shimada, Shikano, & Merila, 2011; Whitehead et al., 2012; Yan, Wang, & Zhao, 2013). An additional eight transcripts belonging to the claudin family of tight‐junction proteins also have nonsynonymous SNPs. The expansion of this gene family in the European sea bass is suggested to have allowed it to adapt to variable salinities (Tine et al., 2014), and this is also one of the largest gene families in the pejerrey genome (Campanella, 2014).

The transition from marine to fresh water involves ecological changes beyond the salinity gradient. Calcium concentrations are higher in marine water than freshwater, which has been implicated in bony plate (armor) loss in sticklebacks that have transitioned to fresh water (Bell et al., 1993; Spence et al., 2012). *Odontesthes* species have no bony armor, but all teleosts absorb environmental calcium for bone formation (Simmons, 1971). Bone morphogenic protein 3 (BMP3) was expressed at least five times higher in *O. argentinensis* than any *O. bonariensis* treatment, suggesting that pejerrey fish also experience differences in bone growth in these different environments. Heat shock protein 90‐beta also was both differentially expressed and contained a nonsynonymous SNP that distinguished the two species. While in the gill, this gene is probably responsible for mediating responses to changes in temperature and other stressors. Otherwise, over expression of this gene has been reported in ovary during the thermo‐labile sex‐differentiation period in larval *O. bonariensis* (Fernandino et al., 2011).

### 4.2 Plasticity in *O. bonariensis*

The transcriptomic response measured through gene expression of *O. bonariensis* individuals from Lake Chascomús acclimated to brackish water does not approach the natural state of an *O. argentinensis* marine fish, suggesting that the expression differences we see between the two species are not due to plasticity. While marine salinity (30 ppt) can be lethal to *O. bonariensis*, this species is more comfortable at brackish salinities (Tsuzuki, Aikawa, Strüssmann, & Takashima, 2000; Tsuzuki et al., 2001), and is reported to have a relatively euryhaline gill structure (Vigliano et al., 2006). Given this, we might expect some plasticity in the response of the gills of *O. bonariensis* to changes in salinity, but they have evidently lost the ability to move completely between salinities, and due to this we are unable to evaluate the changes in expression for this freshwater fish in a marine environment. We are able to determine that when exposed over several days to a higher salinity (15 ppt), relatively few transcripts of *O. bonariensis* show a similar expression pattern to *O. argentinensis*. In contrast, the expression profiles of *O. argentinensis* and *O. bonariensis* are largely different, and have more than 3,000 differentially expressed transcripts that distinguish them.

However, a few transcripts show a plastic response in salinity in *O. bonariensis,* that is similar in expression to a marine *O. argentinensis*. Notably, several NEDD4 ubiquitin ligases are expressed more highly in *O. argentinensis*, which interact with epithelial sodium channels, and may be a response to changes in osmolality (Fiol & Kültz, 2007), and one isoform was also expressed by *O. bonariensis* when exposed to brackish water. The higher proportion of these NEDD4 ubiquitin ligases expressed in *O. argentinensis* may be due to these fish often entering estuaries, including the Mar Chiquita estuary, which is near Mar del Plata, where these individuals were collected, even though they were collected in seawater (González‐Castro et al., 2016). Although production of *O. argentinesis* larvae for aquaculture is well established (Sampaio 2006), no success has been achieved to transfer to the laboratory adult *O. argentinensis* captured at sea to test its ability to tolerate freshwater conditions (González‐Castro pers. com.), precluding common garden experiments involving this species.

### 4.3 Candidate genes in immune response

The marine–freshwater boundary strongly structures microorganism communities (Logares et al., 2009) and, while teleost microbiomes have not been extensively characterized, salinity has been shown to influence microbiota in fishes (Lokesh & Kiron, 2016; Schmidt et al., 2015). We found differentially expressed genes between *O. argentinensis* and all *O. bonariensis* to be enriched for the immune response GO term, suggesting that the marine‐to‐freshwater transition may also require changes to the immune system. Within this differentially expressed set, we annotated six pathogen‐detecting MHC I and two MHC II transcripts, and additionally six MHC II transcripts contain nonsynonymous SNPs differentiated between marine and freshwater in our dataset. These genes have been linked to habitat diversity and speciation in fishes (Malmstrøm et al., 2016), and differences between marine and freshwater pathogens and parasites are likely being reflected in our *Odontesthes* system, although these genes also play a role in mate selection in the wild (Bernatchez & Landry, 2003).

In addition to the MHC transcripts, nine lectin transcripts were differentially expressed between these two species. Lectins are pathogen‐recognition molecules important in the innate immune system, which is thought to be the primary defense against pathogens in fish (Vasta et al., 2011), and of which the C‐type lectin is known to be differentially expressed between anadromous and resident populations of trout (Boulet, Normandeau, Bougas, Audet, & Bernatchez, 2012). Four different C‐type lectin isoforms are expressed differently between these species of silversides, which is one strategy for lectins to increase the repertoire of pathogens to which they bind (Bianchet, Odom, Vasta, & Amzel, 2002; Suzuki, Tasumi, Tsutsui, Okamoto, & Suetake, 2003). Thus, the different repertoires of lectins expressed by marine and freshwater fish suggest a possible plastic response to different pathogen regimes between environments.

Some genes related to immune function also are involved in cell signaling, including interleukin 2 and 12 receptors that were differentially expressed here. Interleukin 12 receptors act as a cytokine, that is released by antigen‐presenting cells to stimulate many immune‐related cells in response (Wang & Secombes, 2013). However, genes involved in cell signaling can also act in stress responses to other environmental factors like salinity. Interleukin‐8, also differentially expressed between species, may play a role in immune function as well as osmotic signaling (Kültz, 2012). Whether these genes play a primarily osmotic‐sensing role or immunological role in the wild is difficult to disentangle here, but their complex role in stress responses make them all the more relevant to overcoming the challenge of adapting to a new environment.

### 4.4 Microbiome differentiation

There was substantial individual variation in the gill bacterial communities of wild *O. argentinensis* and *O. bonariensis*, which were somewhat differentiated between marine and freshwater for wild‐collected individuals (Figure 3), further highlighting the different microbial regimes. However, the microbiome of laboratory individuals showed a very different composition that did not appear to be impacted by changing the tank salinity. Fewer than thirty taxa identified by PhyloSift were shared across all individuals, which suggest that there is not a consistent core *Odontesthes* gill microbiome across the natural and experimental environments.

Marine and freshwater microbial taxa are often from phylogenetically distinct lineages and have made relatively few transitions between these environments (Logares et al., 2009). This is a challenge that marine fish will have to overcome as they adapt to freshwater. Studies have documented substantial overturn of the microbiome in different fish tissues as the salinity is increased (Lokesh & Kiron, 2016; Schmidt et al., 2015), although this is frequently accomplished in the laboratory. Our data show that the microbial community observed in the laboratory are likely to be very different from the microbes that inhabit fish the in the wild, although amplicon‐based sequencing would reveal a more complete picture of the microbial communities as a whole. This may hinder future inferences about host‐microbe interactions conducted in the laboratory.

## 5 CONCLUSIONS

Mitochondrial markers have previously suggested very little divergence between *O. argentinensis* and *O. bonariensis*, despite their occupation of highly divergent habitats. This preliminary analysis identified more than 3,000 transcripts that are differentially expressed between these species, and additionally transcripts with nonsynonymous coding SNPs that we propose as candidate genes for this habitat transition and as the basis for divergent ecological adaptation in these species. The broad range of physiological functions associated with these genes suggests that salinity is only one of many abiotic and biotic factors that are driving this divergence. Laboratory experiments focusing on qPCR validation of a few candidate genes have certainly shed light on some of the physiological requirements to make a shift between salt and freshwater, but relatively few studies look at gene expression in natural populations of fishes. Our results suggest that there are both gene expression and sequence divergence differences in these sister species that are driven by the different environments that they inhabit, and that their speciation may be driven by adaptation to different local conditions.

## CONFLICT OF INTEREST

None declared.

## AUTHOR CONTRIBUTIONS

LH and GO designed the research. GS, MG, and JM collected gill samples for the project and carried out laboratory experiments. LH carried out the laboratory work, LH, BN, JB, and GO analyzed the data, and wrote the paper. All authors contributed to the final version of the manuscript.

## DATA ACCESSIBILITY

Sequence reads are deposited in the NCBI Sequence Read Archive (SRA), under BioProject PRJNA311227. Transcriptome assembly and annotation files, scripts for EBSeq, and VCF files for SNP calls can be accessed on Figshare, https://doi.org/10.6084/m9.figshare.4937684.
